# Ambient light drives verteporfin-induced protein cross-linking during standard laboratory sample processing

**DOI:** 10.1371/journal.pone.0358654

**Published:** 2026-09-21

**Authors:** Konstantinos G. Baroutis, Victor San Martin C. Corrêa, Dimitrios Ntentakis, Toshio Narimatsu, Evangelos S. Gragoudas, Joan W. Miller, Lucy H. Young, Demetrios G. Vavvas

**Affiliations:** Department of Ophthalmology, Retina Service, Ines and Frederick Yeatts Lab in Retina Research, Massachusetts Eye and Ear, Harvard Medical School, Boston, Massachusetts, United States of America; Regeneron Pharmaceuticals Inc, UNITED STATES OF AMERICA

## Abstract

Verteporfin (VP) is widely used as a light-independent pharmacologic inhibitor of the YAP-TEAD transcriptional complex, despite its potent photosensitizing properties. Here we show that VP-associated high–molecular–weight complexes and apparent depletion of key proteins on immunoblots can be substantially driven by ambient-light exposure during post-lysis handling, more than by light during cellular treatment. In this study, the hypothesis is tested independently using MEL270, HEK293, and MCF-7 cells treated with therapeutic and supra-therapeutic concentrations of VP under four systematically varied light/dark conditions. The results demonstrate that high-molecular-weight complex (HMWC) formation for p62, DIAP1, ROCK1, YAP, and phospho-YAP is driven principally by light exposure during sample processing rather than during cell treatment, occurs in cell-free lysates within 1 hour of ambient-light exposure, and is markedly attenuated by both N-acetylcysteine and L-histidine, consistent with contributions from both radical-mediated and singlet oxygen-mediated photochemistry. Notably, YAP and phospho-YAP are highly susceptible to photo-cross-linking, whereas transcription enhancer factor 1 (TEF1)/TEAD1 remains comparatively resistant, a pattern that mimics selective YAP-TEAD inhibition on standard immunoblots. In a single-experiment CCK-8 assay, viability was markedly lower under light than in darkness at 24 hours, an observation consistent with the immunoblot findings but requiring independent replication. These findings support the light-artifact hypothesis and may have implications for how preclinical data on VP are interpreted in the design of clinical investigations.

## Introduction

Verteporfin (VP) was approved by the U.S. Food and Drug Administration in 2000 for the treatment of subfoveal choroidal neovascularization associated with age-related macular degeneration (AMD) [1, 2]. In the first step of photodynamic therapy (PDT), liposomal VP (Visudyne) is administered intravenously and, after a quarter of an hour, accumulates in the retinal vasculature and is activated by a semiconductor red-light diode laser at 689 nm [2], generating reactive oxygen species [1], which cause thrombotic occlusion of the targeted neo-vessels [3]. Although intravitreal injections targeting vascular endothelial growth factor have become the first-line treatment of neovascular AMD, PDT with VP remains an important treatment for polypoidal choroidal vasculopathy, chronic central serous chorioretinopathy, and other chorioretinal conditions [3,4].

Moreover, VP is a small-molecule inhibitor of the Yes-associated protein (YAP)-TEA domain (TEAD) transcriptional interaction, thereby extending its utility beyond PDT [5]. YAP is a central effector of the Salvador-Warts-Hippo signaling pathway, also known as the Hippo pathway [6], which controls organ size, tissue homeostasis, and cell proliferation and, when dysregulated, drives the transcription of pro-proliferative and anti-apoptotic genes in many cancers [7]. Initial studies claimed that VP directly binds to YAP and inhibits the YAP-TEAD interaction, independently of light activation [8]. Subsequent studies have indicated further mechanisms, including sequestration of YAP in the cytoplasm via 14-3-3σ [9,10]. This has led to extensive preclinical studies of VP as the prototypical pharmacological YAP inhibitor in a variety of cancers, including glioblastoma, breast, bladder, and pancreatic cancers [11–14]. Here, we tested the hypothesis that ambient light exposure during routine sample processing is a primary determinant of VP-associated protein cross-linking and apparent YAP depletion.

VP was also identified as an autophagy inhibitor. The selective autophagy receptor p62/SQSTM1 is covalently cross-linked into high-molecular-weight complexes (HMWCs) [15,16]. This protein aggregation extends well beyond p62: Zhang et al. reported broad-spectrum proteotoxicity independent of YAP1 expression [17], and Condurat et al. showed that VP-induced proteotoxicity was not abolished in CRISPR-mediated YAP/TAZ-knockout cells [18]. VP was classified as a covalent protein polymerizer with non-specific cross-linking activity [19]. In vivo data on VP-induced antiproliferative effects were equivocal [20,21]. This major discrepancy between high in vitro cytotoxic activity and weak in vivo effects of VP raises a critical mechanistic question: is the effect attributed to VP in cell culture confounded by an uncontrolled experimental variable?

The photochemistry of porphyrin derivatives provides a well-established explanation for this discrepancy. Benzoporphyrins generate singlet oxygen upon exposure to visible light. Singlet oxygen oxidizes susceptible amino acids such as methionine, histidine, tryptophan, cysteine, and tyrosine, resulting in covalent cross-links between polypeptides [22,23]. Fancy and Kodadek took advantage of this photochemistry to map protein-protein interactions and showed that even a short exposure to light in the presence of porphyrin-treated samples leads to extensive covalent oligomerization [24,25]. In a recent study, Jiang et al. showed that singlet oxygen also oxidizes disulfide bonds, resulting in the formation of thiosulfinate intermediates that cross-link polypeptides [26]. Together, these data suggest that the high-molecular-weight complexes, apparent YAP depletion, and cytotoxic effects observed in VP-treated cells may be photochemical artifacts rather than a true pharmacological effect of VP.

This hypothesis was tested directly in a study, which reported that VP-induced formation of cross-linked oligomers and high-molecular-weight complexes is mediated by light and causes cellular toxicity. Its retraction [27] removed from the peer-reviewed literature the primary experimental support for the light-artifact hypothesis, leaving this hypothesis resting only on the general photochemistry of porphyrin derivatives, thus requiring independent re-evaluation [22–26]. We do not rely on any of its data here.

Since VP is now being tested in clinical trials as a systemic anticancer drug [28], and the next-generation TEAD-specific inhibitors are also being tested in clinical trials [29], it has become urgent to clarify whether the reported effects of VP on YAP and other targets are real pharmacological responses or merely photochemical artifacts.

In the present study, we directly tested this hypothesis by treating three cell lines with VP at therapeutic and supra-therapeutic concentrations under four systematically varied light/dark conditions. The results of this study, which were obtained independently, provide new primary evidence for a hypothesis that currently lacks such evidence and may have an impact on how preclinical VP data are generated and interpreted, including the design of ongoing clinical investigations.

## Results

### Ambient light during sample processing drives VP-dependent HMWC formation

**Fig 1:**
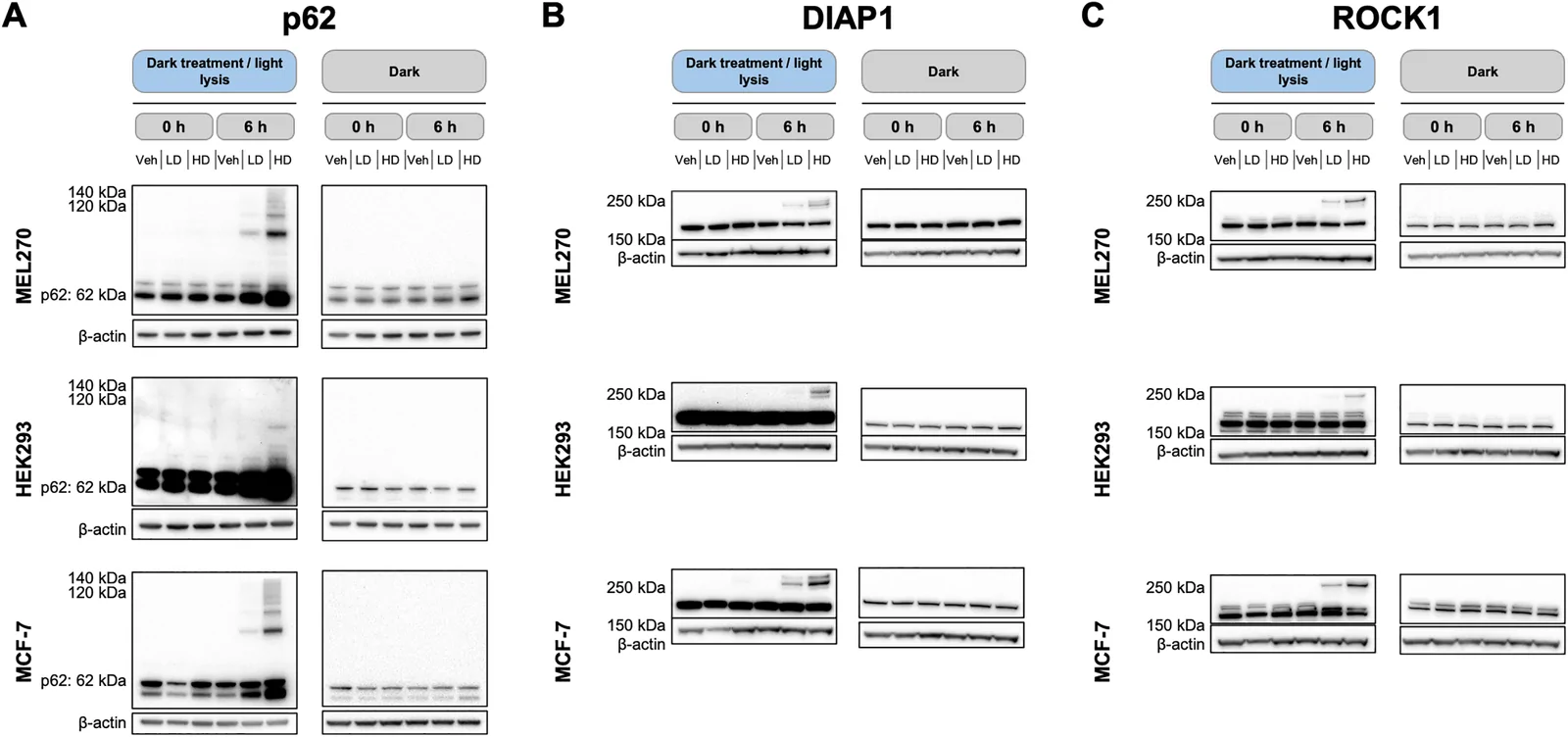
Ambient light during sample processing drives VP-dependent HMWC formation. Western blots of (A) p62, (B) DIAP1, and (C) ROCK1 in MEL270, HEK293, and MCF-7 cells treated with vehicle, low-dose (LD; 1.25 µg/mL), or high-dose (HD; 7.5 µg/mL) VP for 0 or 6 h. Samples underwent dark treatment/light processing (condition ii) or darkness throughout (condition iv). High-molecular-weight complexes (HMWCs) appeared in light-processed VP samples but were absent under darkness. Vehicle lanes are VP-free light controls. β-actin is the loading control.

To determine whether ambient light mediates VP-induced protein cross-linking, MEL270, HEK293, and MCF-7 cells were treated with vehicle, low-dose (LD; 1.25 µg/mL), or high-dose (HD; 7.5 µg/mL) VP under four systematically varied light/dark conditions. Western blot analysis of p62, DIAP1, and ROCK1 revealed that HMWC formation was determined principally by light exposure during the sample-processing phase. Cells treated in darkness and subsequently lysed under ambient light (condition ii: dark treatment/light lysis) exhibited prominent HMWC bands for all three proteins, whereas matched samples processed entirely in darkness (condition iv: complete dark) displayed only monomeric bands at the expected molecular weights (Fig 1). The p62 immunoblots showed the most conspicuous cross-linked species, migrating above 120 kDa, with a concomitant reduction in the monomeric 62-kDa band. DIAP1 and ROCK1 similarly exhibited HMWC bands above 150 kDa exclusively in samples processed under ambient light. This pattern was consistent across all three cell lines. β-actin loading controls confirmed equivalent protein loading across all conditions. Vehicle-treated samples carried through the identical ambient-light workflow showed no HMWC formation and preserved monomeric bands for every protein examined, indicating that ambient light alone, in the absence of VP, does not produce cross-linking.

### VP-induced cross-linking occurs in cell-free lysates and accumulates over hours

**Fig 2:**
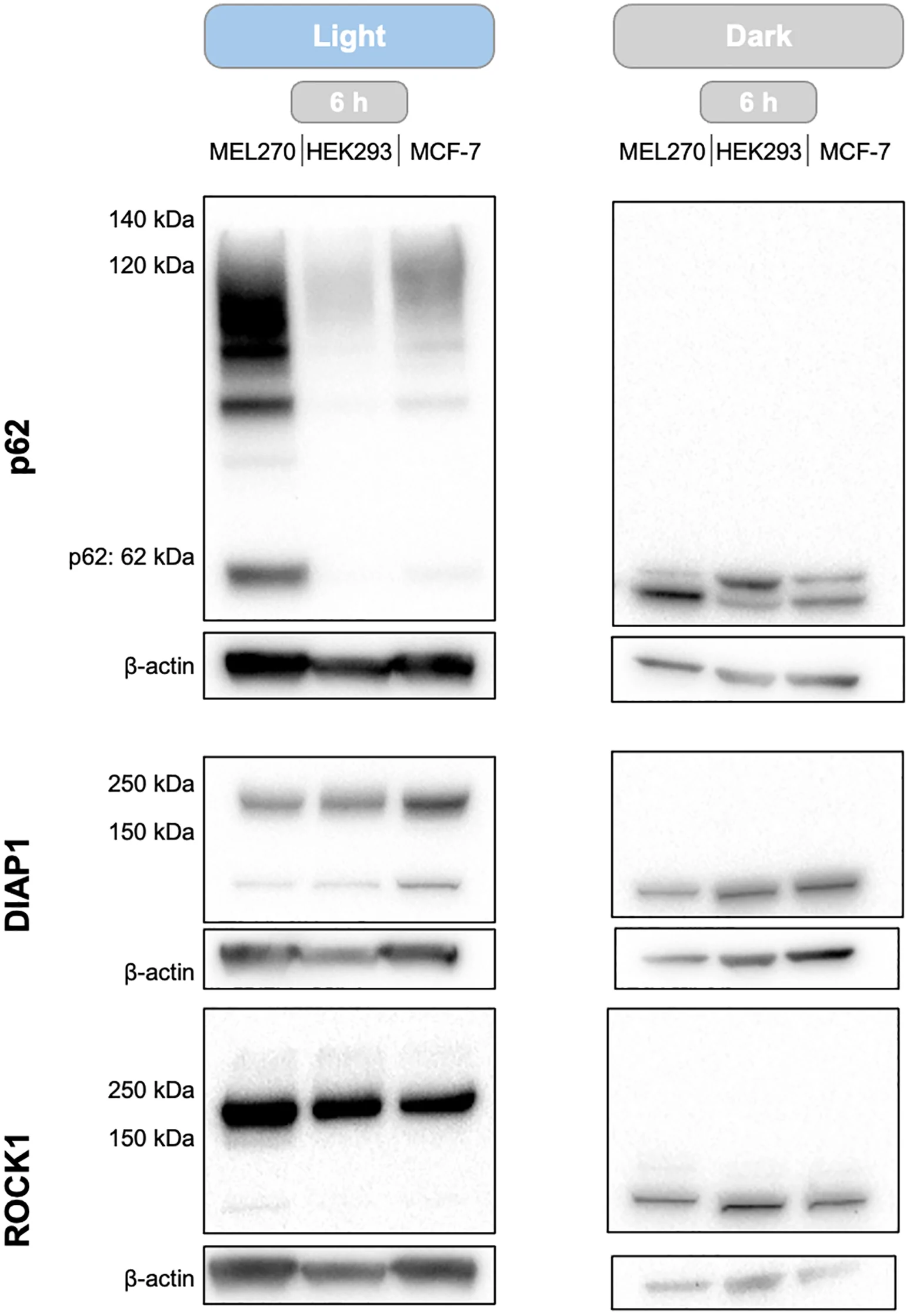
VP induces protein cross-linking in cell-free lysates. Lysates from untreated MEL270, HEK293, and MCF-7 cells were spiked with high-dose (HD; 7.5 µg/mL) VP and incubated for 6 h under ambient light or near-darkness. High-molecular-weight complexes of p62, DIAP1, and ROCK1 formed under light but not in darkness, demonstrating that cross-linking can occur after lysis without intact cellular machinery. β-actin is the loading control.

**Fig 3:**
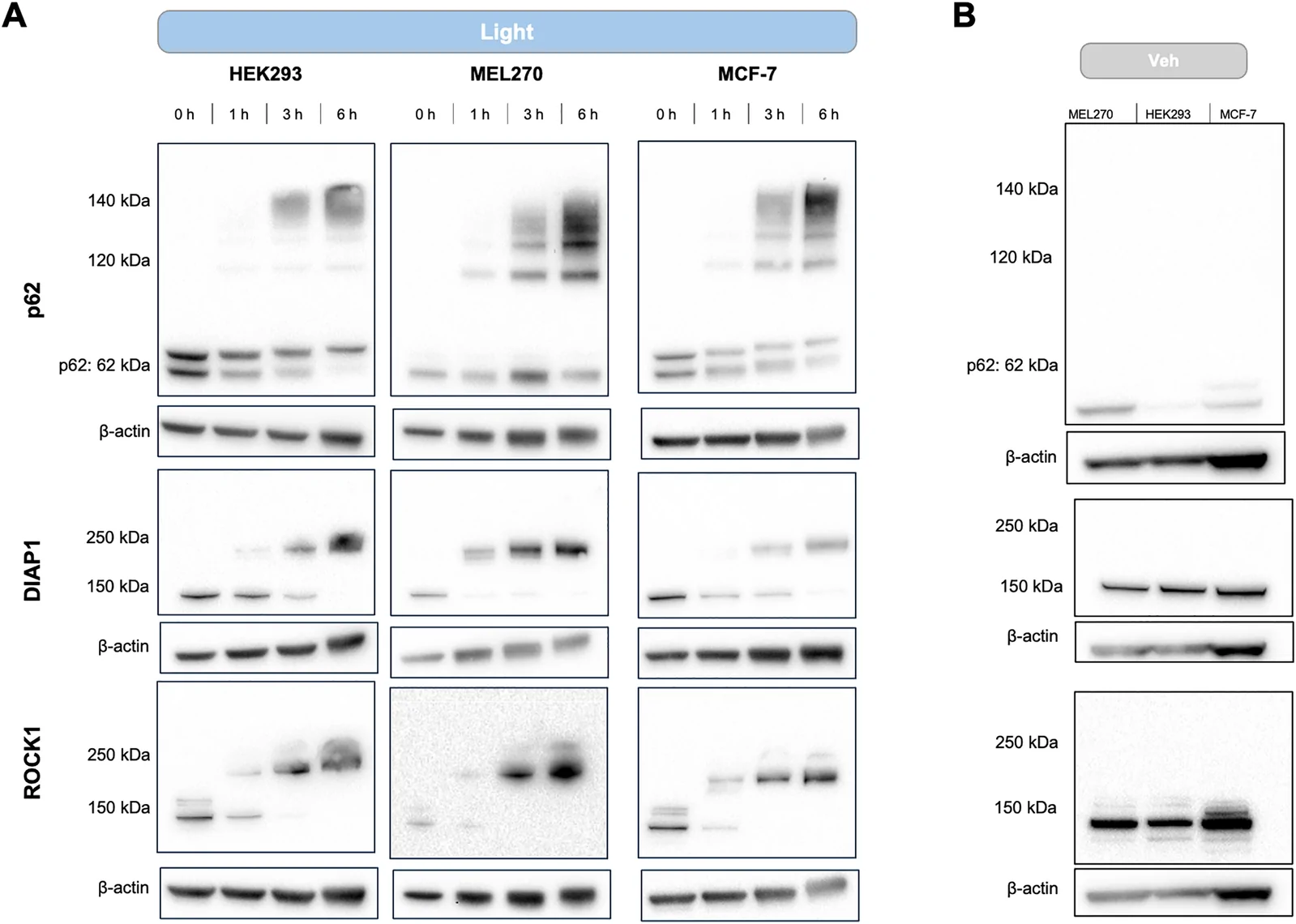
Cross-linking in cell-free lysates accumulates over time and requires VP. (A) Lysates from HEK293, MEL270, and MCF-7 cells were spiked with high-dose (HD; 7.5 µg/mL) VP and exposed to ambient light for 0, 1, 3, or 6 h. High-molecular-weight complexes of p62, DIAP1, and ROCK1 first appeared at 1 h and increased thereafter. (B) Vehicle-treated lysates processed under identical light conditions showed no complexes and preserved monomeric bands, serving as VP-free light controls. β-actin is the loading control.

To establish that VP-induced cross-linking does not require intact cellular machinery, lysates from untreated MEL270, HEK293, and MCF-7 cells were spiked with HD VP (7.5 µg/mL) and incubated on ice. After 6 hours under ambient light, prominent HMWC bands were observed for p62, DIAP1, and ROCK1 in all three cell lines (Fig 2). Matched lysates incubated in near-darkness for the same duration showed no HMWC formation, with only monomeric bands detected. In a time course of light-exposed lysates, cross-linked species were absent immediately after VP addition (0 hours), first detectable at 1 hour, and increased in intensity through 6 hours (Fig 3A). Vehicle-treated samples from all three cell lines, carried through the same ambient-light workflow with no VP added at any stage, showed no HMWC formation and preserved monomeric p62, DIAP1, and ROCK1 (Fig 3B), confirming that ambient light in the absence of VP does not generate cross-linked species. The same complexes form when intact cells are treated with VP before lysis and the lysates are subsequently processed under ambient light (Fig 1). These findings demonstrate that VP-induced protein cross-linking is a photochemical reaction that occurs in the post-lysis protein milieu and does not require active cellular processes.

### Singlet oxygen and free radical scavengers attenuate VP-induced cross-linking

**Fig 4:**
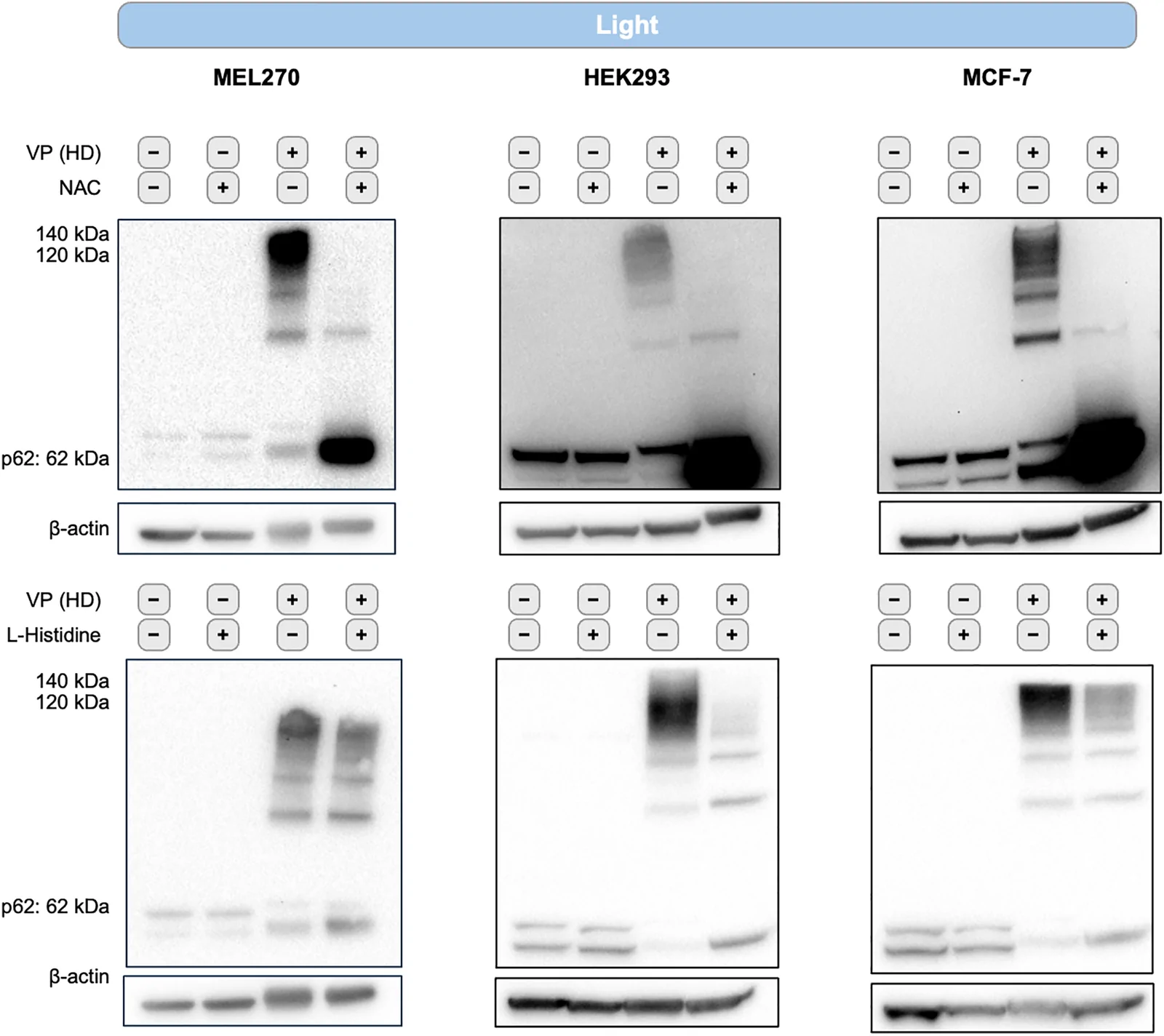
NAC and L-histidine attenuate VP-induced p62 cross-linking. MEL270, HEK293, and MCF-7 homogenates were pre-incubated for 30 min with N-acetylcysteine (NAC; 100 mM; upper panels) or L-histidine (7.5 mM; lower panels), followed by high-dose (HD; 7.5 µg/mL) VP and 6 h of ambient-light exposure. Both scavengers reduced high-molecular-weight complex formation and preserved monomeric p62; attenuation was greater with NAC. VP-negative lanes underwent the same light workflow. β-actin is the loading control.

To identify the reactive species responsible for cross-linking, cell homogenates were pre-incubated with the radical scavenger N-acetylcysteine (NAC; 100 mM) or the singlet oxygen quencher L-histidine (7.5 mM) for 30 minutes before addition of HD VP (7.5 µg/mL) and incubation under ambient light for 6 hours. NAC pre-treatment markedly reduced p62 HMWC formation in MEL270, HEK293, and MCF-7 lysates, leaving only faint residual high-molecular-weight signal with a preserved monomeric 62-kDa band (Fig 4, upper panels). L-histidine pre-treatment also reduced HMWC formation, although residual high-molecular-weight signal remained in all three cell lines and the reduction was less complete than with NAC (Fig 4, lower panels). In both cases, the monomeric p62 signal was maintained, indicating that the scavengers limited cross-linking rather than promoting non-specific protein degradation. These results are consistent with the involvement of both Type II (singlet oxygen-mediated) and Type I (radical-mediated) photochemical pathways in VP-induced cross-linking.

### Methionine oxidation parallels light-dependent cross-linking

**Fig 5:**
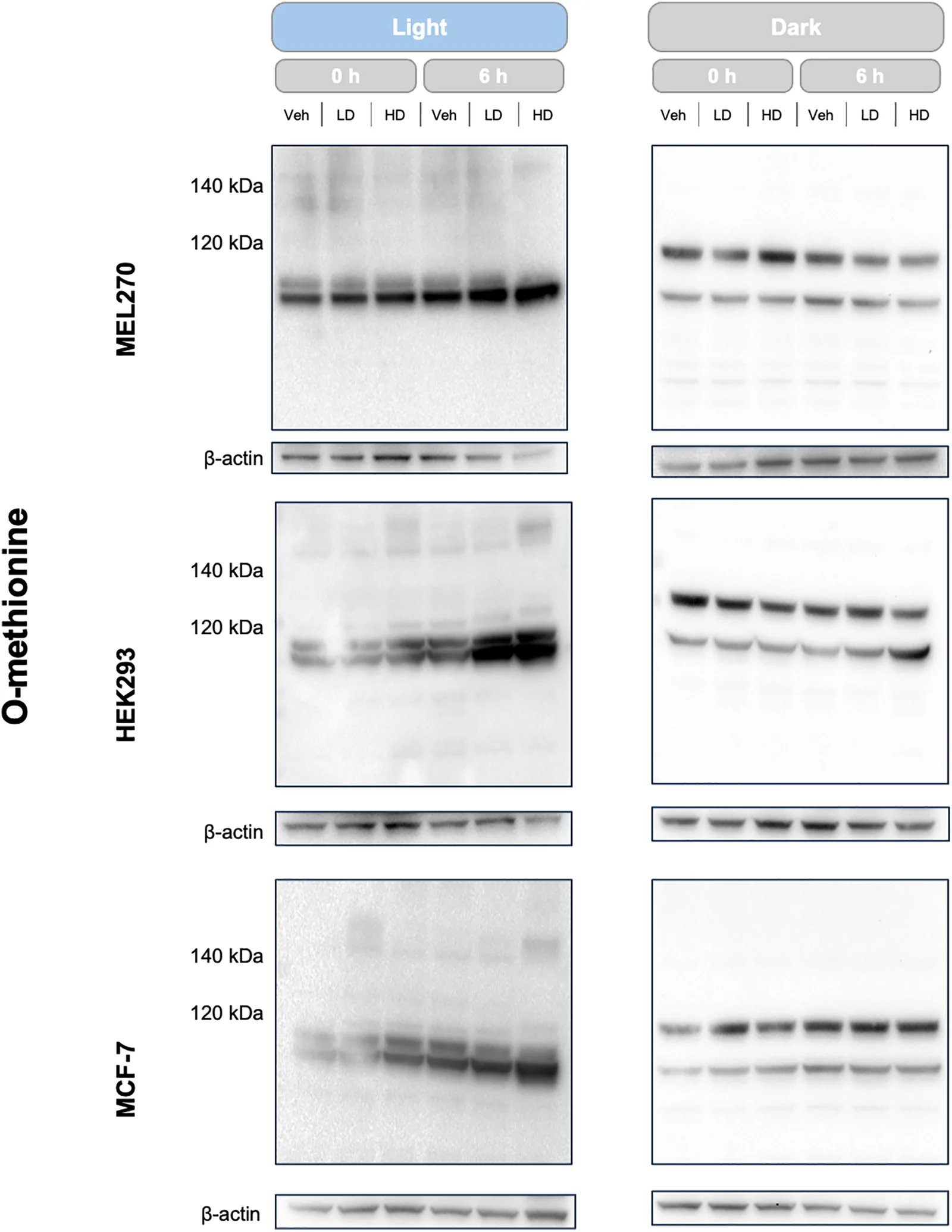
Light-dependent methionine oxidation parallels VP-induced cross-linking. Anti-methionine sulfoxide immunoblots of MEL270, HEK293, and MCF-7 cells treated with vehicle, low-dose (LD; 1.25 µg/mL), or high-dose (HD; 7.5 µg/mL) VP for 0 or 6 h under ambient light throughout (condition i) or darkness throughout (condition iv). Immunoreactivity at approximately 120–140 kDa generally increased with VP dose and duration under light and was lower in dark-processed samples. Vehicle lanes are VP-free light controls. β-actin is the loading control.

Immunoblotting with an anti-methionine sulfoxide antibody revealed light-dependent methionine oxidation across all three cell lines (Fig 5). Under ambient light, immunoreactive bands in the 120–140-kDa range increased in intensity with VP dose and treatment duration. These signals were more prominent in VP-treated than in vehicle-treated samples, although light-exposed vehicle samples also displayed detectable methionine sulfoxide immunoreactivity. Samples processed in near-darkness manifested minimal methionine sulfoxide signal regardless of VP treatment. The concordance between methionine oxidation and HMWC formation indicates that singlet oxygen was generated under these conditions. Methionine is one of several residues attacked by singlet oxygen, alongside tryptophan, tyrosine, histidine and cysteine, and we did not determine which residues carry the covalent linkages responsible for HMWC formation.

### YAP and phospho-YAP undergo light-dependent cross-linking; TEF1 is comparatively resistant

**Fig 6:**
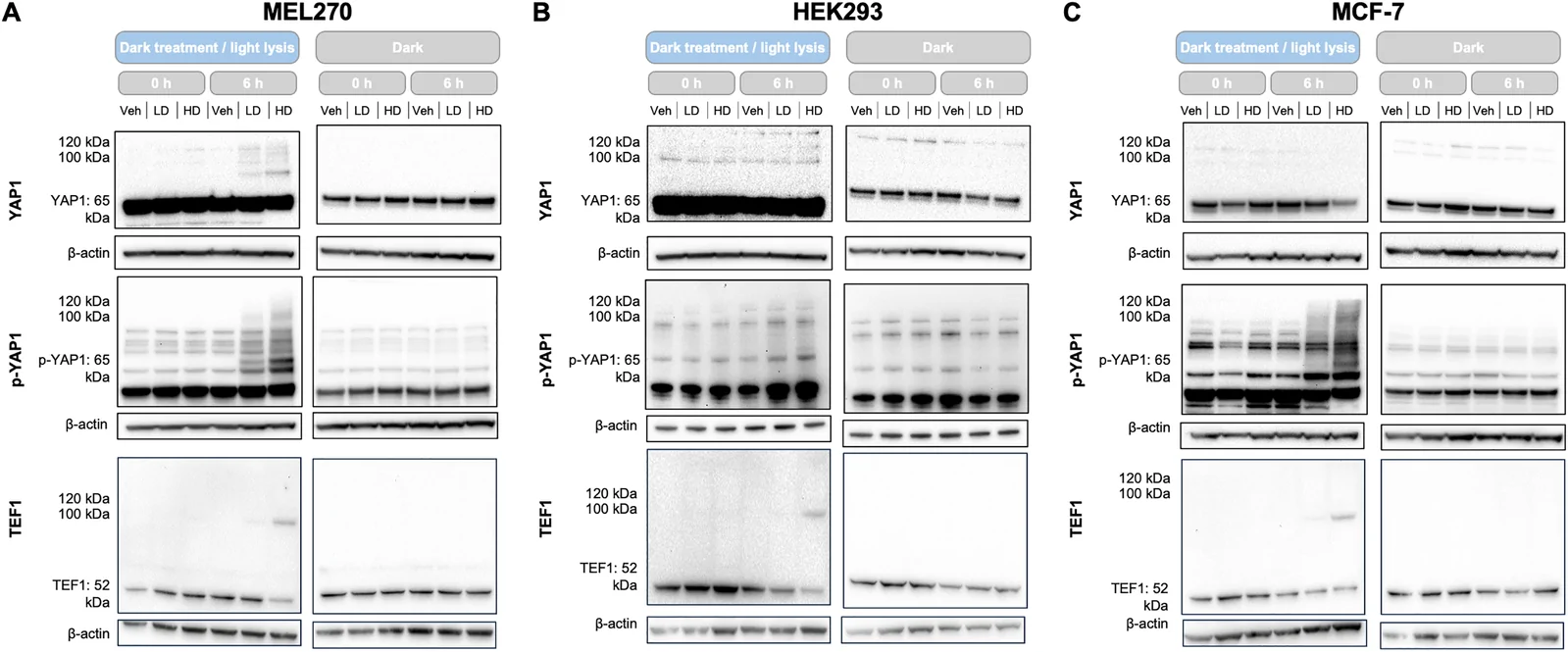
YAP and phospho-YAP undergo light-dependent cross-linking, whereas TEF1/TEAD1 is comparatively resistant. Western blots of YAP, phospho-YAP (Ser127), and TEF1/TEAD1 in (A) MEL270, (B) HEK293, and (C) MCF-7 cells treated with vehicle, low-dose (LD; 1.25 µg/mL), or high-dose (HD; 7.5 µg/mL) VP for 0 or 6 h. Samples underwent dark treatment/light processing (condition ii) or darkness throughout (condition iv). Light-processed VP samples showed YAP and phospho-YAP high-molecular-weight complexes with reduced monomeric bands, whereas TEF1/TEAD1 remained largely stable. β-actin is the loading control.

Given the widespread use of VP as a purported YAP-TEAD inhibitor, we examined whether YAP, phospho-YAP (Ser127), and TEF1/TEAD1 are susceptible to light-dependent cross-linking. Under dark treatment/light lysis conditions, YAP exhibited HMWC bands and reduced monomeric band intensity in a dose- and time-dependent manner across MEL270, HEK293, and MCF-7 cells (Fig 6). Phospho-YAP (Ser127) displayed a parallel pattern of HMWC formation with diminished monomeric signal under light exposure. Under near-darkness, both YAP and phospho-YAP monomeric bands were preserved at levels comparable to vehicle-treated controls, with no detectable HMWC formation.

TEF1/TEAD1 exhibited substantially less HMWC formation than either YAP or phospho-YAP. The monomeric TEF1 band remained largely stable across doses and time points, with reduced monomeric signal and a discrete higher-molecular-weight species evident only at the high dose at 6 hours under light (Fig 6). This differential susceptibility suggests that structural determinants governing cross-linking vulnerability vary among proteins, with TEF1 being comparatively, though not entirely, resistant to VP-induced photochemical modification.

### Light during treatment contributes less to HMWC formation than light during processing

**Fig 7:**
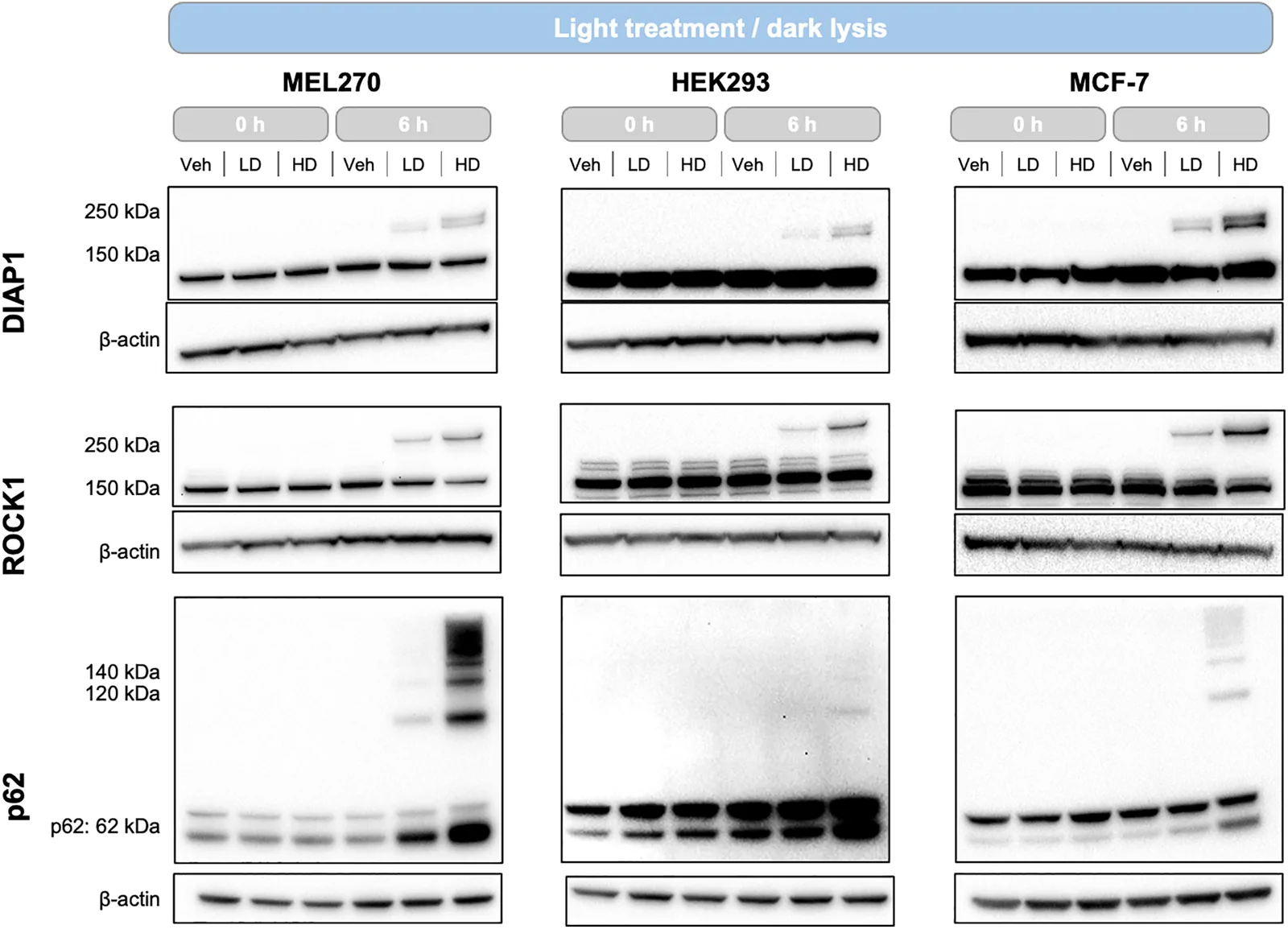
Light during treatment contributes less to HMWC formation than light during processing. Western blots of DIAP1, ROCK1, and p62 in MEL270, HEK293, and MCF-7 cells treated with vehicle, low-dose (LD; 1.25 µg/mL), or high-dose (HD; 7.5 µg/mL) VP for 0 or 6 h under light treatment/dark processing (condition iii). High-molecular-weight complex formation was attenuated relative to dark treatment/light processing (condition ii; Fig 1), indicating that post-lysis illumination is the stronger determinant of cross-linking. β-actin is the loading control.

When cells were treated with VP under ambient light but lysed and processed in near-darkness (condition iii: light treatment/dark lysis), HMWC formation for DIAP1, ROCK1, and p62 was attenuated relative to conditions in which lysis occurred under light (Fig 7). This result indicates that, although some cross-linking may occur during the treatment phase, light exposure during the lysis and sample-processing steps is the primary determinant of HMWC formation.

### VP combined with ambient light reduces cell viability in a dose- and time-dependent manner

**Fig 8:**
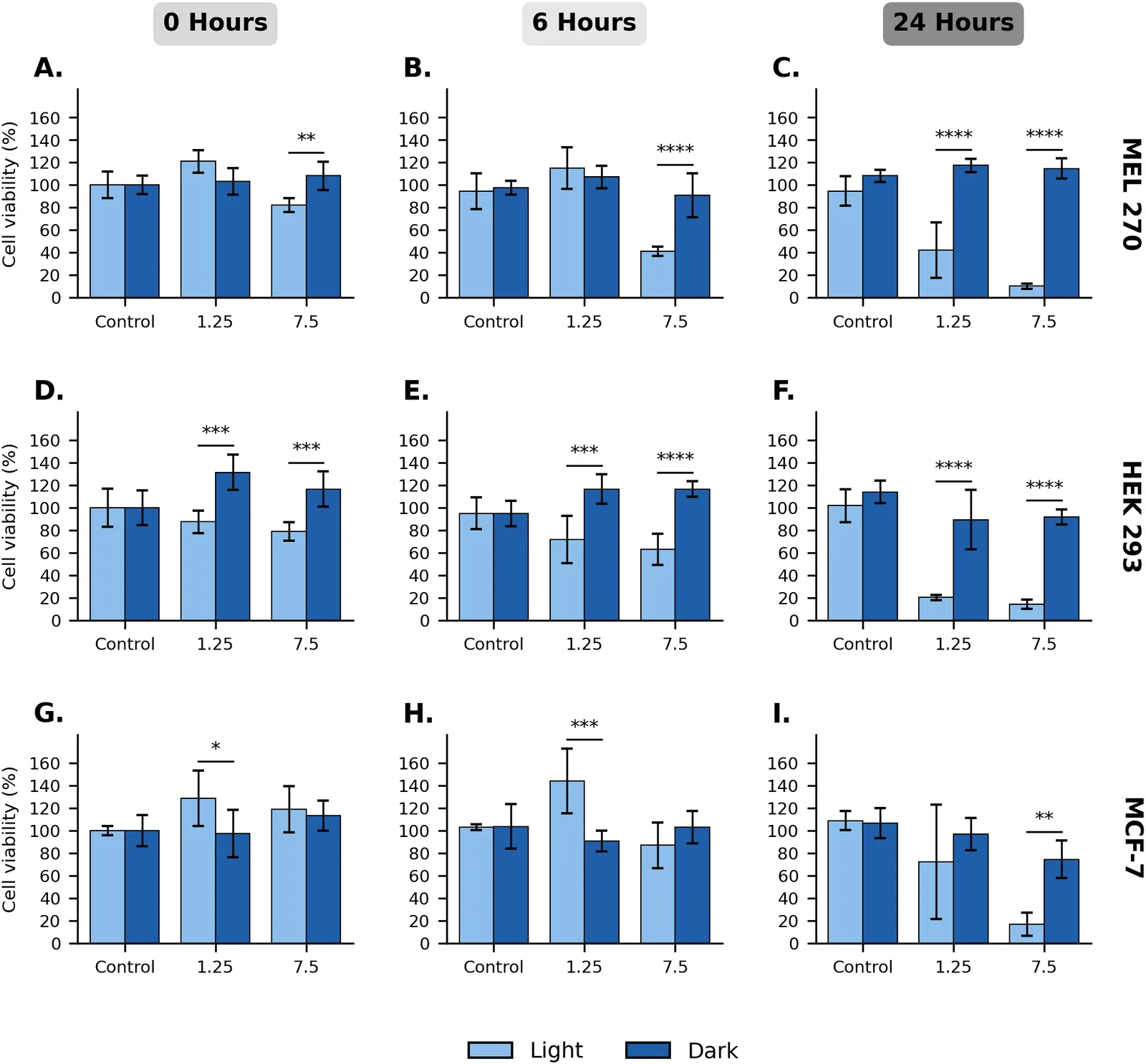
Light-dependent changes in cell viability after VP treatment. CCK-8 viability in MEL270 (A–C), HEK293 (D–F), and MCF-7 (G–I) cells treated with vehicle, low-dose (LD; 1.25 µg/mL), or high-dose (HD; 7.5 µg/mL) VP under ambient light or darkness for 0 h (<5 min), 6 h, or 24 h. Values are percentages of vehicle control and mean ± SD from six technical replicate wells in one experiment. Two-way ANOVA with Tukey HSD assessed within-plate differences; brackets compare light versus dark within dose. *p < 0.05, **p < 0.01, ***p < 0.001, ****p < 0.0001.

Cell viability was quantified by CCK-8 assay in MEL270, HEK293, and MCF-7 cells treated with vehicle, LD, or HD VP under ambient light or dark conditions for 0, 6, and 24 hours (Fig 8). Two-way ANOVA (dose × light) was performed for each cell line × time point combination. Vehicle-treated cells maintained viability near 100% across all conditions, confirming that ambient light alone did not impair survival. These viability data derive from a single experiment (six technical replicate wells per condition) and are presented as a hypothesis-generating observation corroborating the immunoblot findings; no conclusion in this study rests on the viability data alone.

At 0 hours (<5 min exposure), dose × light interactions were already detected in MEL270 (η²p = 0.47, p < 0.001) and HEK293 (η²p = 0.37, p = 0.001) but not MCF-7 (p = 0.086; Fig 8A, D, G). By 6 hours, all three cell lines showed interactions (η²p = 0.45–0.50, all p < 0.001; Fig 8B, E, H), with HD VP reducing MEL270 viability to 40.9 ± 4.1% under light versus 90.7 ± 19.5% in darkness (Tukey p < 0.0001). At 24 hours, light-dependent cytotoxicity was pronounced across all cell lines (η²p = 0.24–0.74, all p < 0.02; Fig 8C, F, I): MEL270 viability fell to 10.1 ± 2.5% (HD, light) versus 114.5 ± 9.0% (HD, dark), and HEK293 to 14.2 ± 4.2% versus 91.7 ± 6.8% (Tukey p < 0.0001 for both). Under dark conditions, viability was generally preserved, except for MCF-7 at HD/24 h (74.4 ± 16.7%).

## Discussion

Here we provide evidence that VP-induced cross-linking and the formation of HMWC can be photochemical artifacts resulting from ambient light exposure during routine laboratory procedures. Using three different cell lines (MEL270, HEK293, and MCF-7) and a factorial light/dark experimental design, we tested the hypothesis directly with new reagents, cell passages, and Western blot membranes. This independent verification, combined with the convergent support of decades of porphyrin photochemistry research, provides a firm basis for the artifact hypothesis [22–26].

The requirement for light exposure during sample preparation and the formation of HMWC in cell-free lysates suggest that this is a nonenzymatic process rather than an intracellular signaling pathway. The attenuation of photocross-linking by N-acetylcysteine and L-histidine is consistent with the involvement of both a Type I (radical-mediated) and a Type II (singlet oxygen-mediated) photochemical pathway, consistent with the known photophysics of benzoporphyrin derivatives [30]. Singlet oxygen preferentially oxidizes methionine to a sulfoxide intermediate, which can participate in a secondary cross-linking reaction [22]. Jiang et al. have also shown that singlet oxygen can oxidize disulfide bonds to thiosulfinates, which can cross-link two or more proteins [26]. The accumulation of cross-linked species over the course of hours of illumination is consistent with a porphyrin-sensitized photooxidation in the lysate [24,25, 31]. In the literature, there is a lack of consistency in the description of procedures for protecting the lysate from light. Some investigators have described continuous protection of the lysate from light by covering the lysate with aluminum foil, whereas others have described a procedure analogous to that used for Western blots [10–14]. Combined with the lack of cross-linking in vehicle-treated lanes, which contain no VP but are processed under the same ambient-light conditions, and in lanes that are processed in the dark, which contain the same molecule but are not exposed to light, these results indicate that both light and VP are required for cross-linking and point to photochemically generated reactive oxygen species as the driver of the reaction. A photoinactive analogue of VP would provide a more definitive test of this hypothesis, but we are unaware of a congener of VP that does not generate singlet oxygen, since any change that removes singlet oxygen production would also change the benzoporphyrin chromophore itself.

The differential susceptibility of individual proteins to VP-induced photo-cross-linking has important implications for the use of VP as a YAP-TEAD inhibitor. The monomeric bands of YAP and phospho-YAP were highly prone to HMWC formation in the presence of light in a dose- and time-dependent manner. However, TEF1/TEAD1 was comparatively stable. Consequently, if a sample is treated with VP and then subjected to Western blotting under ambient light, as is the standard procedure, the YAP band will be reduced but the TEAD band will be preserved, a phenotype that is indistinguishable from specific pharmacological interference with the YAP-TEAD interaction. There are two possible structural explanations for the differential susceptibility of YAP and TEAD, although the present data cannot discriminate between them. Singlet oxygen preferentially oxidizes tryptophan, tyrosine, histidine, methionine, and cysteine. Consequently, the susceptibility to photo-cross-linking should be a function of the abundance and solvent exposure of these residues, which is consistent with the methionine sulfoxide immunoreactivity we report. Secondly, YAP is intrinsically disordered across much of its length [32], whereas the TEA domain of TEF1/TEAD1 is a compact three-helix bundle with a homeodomain fold [33]. Consequently, comparable chemistry would be less likely to result in a detectable mobility shift on SDS-PAGE. Both of these explanations are hypotheses rather than conclusions, and only the identification of the cross-linked species by mass spectrometry will distinguish between them. These observations are consistent with Zhang et al., who showed that VP-induced proteotoxicity was independent of YAP1 expression [17], and with Condurat et al., who showed that CRISPR-mediated YAP/TAZ knockout did not abrogate VP-induced proteotoxicity in neuroblastoma cells [18]. The classification of VP as a unique covalent protein polymerizer further supports the notion that its cross-linking activity is non-specific [19], as do its several light-independent biological activities [34].

It is important to be clear about what this study has established. This study measured protein mobility on a Western blot, but it did not measure the transcriptional activity of YAP, the expression of YAP target genes, or the binding of YAP and TEAD. Consequently, this study establishes that ambient light is a confounding factor that can produce a phenotype on a Western blot that is indistinguishable from the selective inhibition of YAP, and it calls for a re-evaluation of earlier studies that relied on Western blots run under uncontrolled lighting. However, this study does not establish that the YAP-pathway effects previously attributed to VP are artifacts.

These data also point to a possible explanation for the well-documented discordance between the in vitro and in vivo effects of VP. For example, Lui et al. reported that VP efficiently degraded YAP and TAZ in cultured melanoma cells but failed to inhibit tumor growth in a mouse model of melanoma at doses matched to human therapeutic exposure [20]. Similarly, Brouwer et al. showed that VP decreased the expression of YAP-pathway proteins in uveal and conjunctival melanoma cells [21]. The light-artifact hypothesis offers a possible explanation for this discordance: tissue culture is performed under ambient light, which activates VP photochemistry, whereas the in vivo tumor microenvironment is shielded from visible light. Thus, at least in part, the apparent in vitro efficacy of VP may be attributable to photochemical proteotoxicity that does not occur under physiological conditions [35].

In the present study, we used the CCK-8 assay to determine cell viability, and our results are consistent with the light-dependent nature of VP’s cellular toxicity. Under dark conditions, cell viability was largely preserved even at the highest dose and the longest exposure time, with the exception of MCF-7 cells at the highest dose at 24 h, which showed a modest reduction in viability to 74.4%. The occurrence of a toxic effect under dark conditions has been reported previously and has been attributed to a low-level oxidative effect that may be augmented by ambient light during the experimental procedure [16]. It should be noted that the immunoblot data were obtained in two independent experiments with three cell lines, whereas the data on cell viability were obtained from a single experiment and are presented as a hypothesis-generating observation that supports, but does not independently establish, the light-dependent nature of VP’s cellular toxicity.

Finally, the results of this study could be used to improve the experimental methods of ongoing clinical trials. In particular, a Phase I/II clinical trial (NCT04590664) is evaluating intravenous VP as a single-agent, light-independent chemotherapeutic for the treatment of recurrent EGFR-mutated glioblastoma [36]. Meanwhile, mechanistically defined, light-independent Hippo pathway tools, such as TEAD palmitoylation/palmitate-pocket inhibitors (e.g., VT3989, IK-930) and direct YAP-TEAD protein-protein interaction inhibitors (e.g., IAG933), that target YAP-TEAD signaling through defined mechanisms, provide the field with pharmacological tools to study Hippo pathway biology without the confounding effects of VP’s photochemistry [37].

There are several limitations to this study. First, the CCK-8 data were derived from a single experiment with six wells per condition on a single 96-well plate, rather than from independent biological experiments. Consequently, the statistical analysis reflects within-plate consistency rather than between-experiment variability. However, several features of the CCK-8 data are internally consistent: (i) the dose- and time-dependent responses were consistent across three different cell lines (MEL270, MCF-7, and HEK293); (ii) a clear dose-response relationship was observed at each time point; and (iii) the CCK-8 data were consistent with the Western blot data from two independent biological experiments. None of these features, however, is a substitute for independent biological experiments. Second, this study did not include direct assays of YAP activity, such as TEAD-reporter assays, quantitation of YAP target gene expression, or measurement of YAP-TEAD binding. Consequently, our conclusions are based only on the Western blot phenotypes. The dark-processed VP arm and the VP-free, light-exposed arm demonstrate that both VP and light are required for cross-linking, but neither can substitute for a photoinactive analog that retains the molecule but removes its photochemistry. Third, VP absorbs a broad range of the visible spectrum, and we did not determine the relative contribution of different wavelengths to cross-linking efficiency. The next step in this study would be to use a monochromatic light source with precise dosimetry. This study was based on cell lines and cell lysates. It did not include patient samples, clinical pharmacodynamics, or clinical outcomes. Consequently, it is not possible to draw conclusions about clinical efficacy. The hypothesis that ambient-light photochemistry contributes to the discrepancy between in vitro potency and in vivo efficacy is a reasonable and testable hypothesis, but it is not proven.

In conclusion, the results of this study suggest that in cell-based studies involving VP, all procedures, such as cell lysis, protein extraction, gel electrophoresis, and membrane transfer, should be performed in dim light or with amber-filtered light that excludes the VP excitation spectrum, to avoid the formation of HMWC of the studied proteins.

## Methods

### Cell lines and reagents

Human embryonic kidney cells (HEK293, CRL-1573) and breast cancer cells (MCF-7, HTB-22) were purchased from the American Type Culture Collection (ATCC, Manassas, VA). Uveal melanoma cells (MEL270) were kindly provided by a laboratory at Massachusetts Eye and Ear Infirmary. RPMI-1640 medium (#30–2001) was obtained from ATCC. Heat-inactivated fetal bovine serum (HI-FBS, #A5670801) and penicillin-streptomycin (#15140122) were from Thermo Fisher Scientific. Dimethyl sulfoxide (DMSO, #4-X) was obtained from ATCC. VP (#SML0534) was purchased from Sigma-Aldrich.

### Cell culture

HEK293 cells were maintained in Dulbecco’s Modified Eagle Medium (DMEM) supplemented with 10% (v/v) HI-FBS, 10 mM HEPES, 100 U/mL penicillin, and 100 µg/mL streptomycin. MCF-7 cells were cultured in RPMI-1640 medium supplemented with 10% (v/v) HI-FBS, 100 U/mL penicillin, and 100 µg/mL streptomycin. MEL270 cells were grown in RPMI-1640 medium supplemented with 10% (v/v) FBS, 10 mM HEPES, 1% MEM vitamin solution, and 1% MEM non-essential amino acids (Thermo Fisher Scientific), with 100 U/mL penicillin and 100 µg/mL streptomycin. All cell lines were maintained at 37°C in a humidified atmosphere containing 5% CO_2_ and passaged every 2–3 days.

### VP treatment and light/dark conditions

Cells were treated with vehicle (DMSO), low-dose (LD; 1.25 µg/mL, corresponding to 1.7 µM) or high-dose (HD; 7.5 µg/mL, corresponding to 10.4 µM) VP for 0 h (immediate removal after addition) or 6 h. To assess the role of ambient light in VP-induced protein cross-linking, four distinct light/dark conditions were employed: (i) ambient light during both cell treatment and all subsequent steps including lysis and electrophoresis; (ii) darkness during cell treatment with ambient light during lysis and electrophoresis; (iii) ambient light during cell treatment with darkness during lysis and electrophoresis; and (iv) near-darkness during all steps, including treatment, lysis, and electrophoresis (S1 Fig). Under dark conditions, all procedures were performed in a dedicated dark room, with minimal indirect red-filtered light sufficient for sample handling. Ambient light exposure consisted of standard overhead illumination from ceiling-mounted troffers fitted with white LED retrofit tubes (Philips LEDtube InstantFit), positioned approximately 1 m above the bench. Bench-level illuminance was measured directly with a digital lux meter (Dr.Meter LX1010B; range 0–100,000 lux) and was 400–500 lux at the sample position, within the range typical of routine laboratory lighting. As phosphor-converted white LED sources, these fixtures emit across the visible range with negligible ultraviolet output; the detailed spectral power distribution was not recorded. Oxygen tension and temperature were not varied between the conditions compared: within each experiment, light-exposed and dark-processed samples were handled in parallel under otherwise identical atmospheric and thermal conditions, so that illumination was the only variable differing between the conditions being compared.

### Post-lysis VP spiking experiments

To determine whether VP can induce cross-linking of proteins in cell lysates independently of cellular activities, lysates from untreated cells were spiked with 7.5 µg/mL VP post-lysis and incubated on ice for up to 6 h in either ambient light or near-darkness.

### Protein extraction and Western blot analysis

Cells were lysed in M-PER Mammalian Protein Extraction Reagent (#78501, Thermo Fisher Scientific) supplemented with cOmplete Mini EDTA-free protease inhibitor cocktail (#11836170001, Roche) and PhosSTOP phosphatase inhibitor (#PHOSS-RO, Roche). Total protein concentration was determined using the Pierce Bradford Protein Assay Kit (#23200, Thermo Scientific). Equal amounts of total protein were loaded onto NuPAGE 4–12% Bis-Tris Mini Protein Gels (#NP0336BOX, Thermo Fisher Scientific) and separated by electrophoresis at 150 V for 1 h 30 min. For dark-condition experiments, electrophoresis was performed in a dark room. Proteins were transferred to PVDF membranes using the eBlot L1 protein transfer system (GenScript). Each protein target was detected on a separate membrane to avoid potential artifacts from antibody stripping and re-probing. Because all target proteins have molecular weights distinct from β-actin (42 kDa), anti-β-actin and anti-target primary antibodies were co-incubated on the same membrane and detected simultaneously with a single HRP-conjugated anti-rabbit IgG secondary antibody; target and loading-control bands were identified by their expected electrophoretic mobilities. Membranes were blocked at room temperature with 5% non-fat dry milk in TBS-T for 1 h (or 5% BSA in TBS-T for 30 min for phosphorylated proteins), then incubated overnight at 4°C with primary antibodies: anti-p62/SQSTM1 (1:1000, #5114, Cell Signaling Technology), anti-DIAP1 (1:1000, #5486, Cell Signaling Technology), anti-ROCK1 (C8F7) (1:1000, #4035, Cell Signaling Technology), anti-YAP (1:1000, #4912, Cell Signaling Technology), anti-phospho-YAP Ser127 (1:1000, #4911, Cell Signaling Technology), anti-TEF1/TEAD1 [EPR3967(2)] (1:1000, #ab133533, Abcam), anti-methionine sulfoxide (1:200, #600166, Cayman Chemical), and anti-β-actin (13E5) (1:2000, #4970, Cell Signaling Technology). After washing, membranes were incubated with HRP-linked anti-rabbit IgG secondary antibody (1:2000, #7074, Cell Signaling Technology) for 30 min at room temperature. All primary and secondary antibodies are listed in S1 Table. Protein bands were visualized using Immobilon Western Chemiluminescent HRP Substrate (#WBKLS0500, Millipore) and imaged on a ChemiDoc XRS+ imaging system (Bio-Rad). All experiments were performed in two independent biological replicates unless otherwise stated.

### Antioxidant and radical scavenger treatment

To investigate the mechanism of VP-induced protein cross-linking, cellular homogenates were pre-treated with NAC (100 mM) or L-histidine (7.5 mM) for 30 min before incubation with high-dose VP (7.5 µg/mL) for 6 h in ambient light. NAC was dissolved in water. Samples were then processed for Western blot analysis as described above.

### Cell viability assay

Cell viability was assessed using the Cell Counting Kit-8 (CCK-8; #NC9261855, Dojindo Molecular Technologies) according to the manufacturer’s protocol. MEL270, HEK293, and MCF-7 cells were seeded at a density of 10,000 cells per well in 96-well plates. The following day, cells were treated with vehicle, 1.25 µg/mL, or 7.5 µg/mL VP under ambient light or in the dark for 0 h (<5 min after washes), 6 h, and 24 h. After the treatment period, 10 µL of CCK-8 reagent was added to each well, and the plate was incubated at 37°C for 4–5 h. Absorbance was measured at 450 nm using a SpectraMax microplate reader (Molecular Devices). Six replicate wells per condition were included on a single 96-well plate (technical replicates from a single experiment). Viability was expressed as a fraction relative to untreated vehicle control cells.

### Statistical analysis

Western blot experiments were performed in two independent biological replicates. For the CCK-8 assay, six technical replicate wells per condition from a single experiment were analyzed. Results are expressed as mean ± standard deviation (SD). CCK-8 data were analyzed using two-way ANOVA (Type II sum of squares; factors: dose and light condition) for each cell line × time point combination (9 ANOVAs), with Tukey’s Honestly Significant Difference (HSD) test for post-hoc pairwise comparisons. Assumption checks included Shapiro–Wilk tests for normality and Levene’s test for homogeneity of variance. Effect sizes are reported as partial eta-squared (η²p) for ANOVA effects and Cohen’s d for pairwise comparisons. A p-value of less than 0.05 was considered statistically significant. Analyses were performed in Python (v3.12) using pandas, scipy, statsmodels (v0.14), and matplotlib. Because the CCK-8 data derive from a single experiment, these analyses quantify differences among wells within that single experiment and do not provide evidence of reproducibility across independent experiments; consistent with this, Levene’s test indicated unequal variances in 4 of the 9 analyses.

## Supporting information

S1 Fig Schematic of the four light/dark conditions. MEL270, HEK293, and MCF-7 cells received vehicle, low-dose (LD; 1.25 µg/mL), or high-dose (HD; 7.5 µg/mL) VP for 0 or 6 h. Illumination was varied independently during treatment and during lysis/electrophoresis, producing: (i) light/light, (ii) dark/light, (iii) light/dark, and (iv) dark/dark. Panels are grouped by treatment illumination, so their displayed order is i, iii, ii, iv. Representative outcomes indicate HMWC formation in conditions i and ii, attenuation in iii, and absence in iv. Cell-free experiments (Figs 2–4) fall outside this design.
(PDF)

S1 Table Primary and secondary antibodies used for Western blot analysis. Listed for each antibody are the host species, working dilution, catalog number, Research Resource Identifier (RRID), and commercial source.
(DOCX)

## References

[1] Kessel D. Photosensitization with derivatives of haematoporphyrin. Int J Radiat Biol Relat Stud Phys Chem Med. 1986;49(6):901–7. doi: 10.1080/09553008514553131 2940198

[2] Photodynamic therapy of subfoveal choroidal neovascularization in age-related macular degeneration with verteporfin: one-year results of 2 randomized clinical trials--TAP report. Treatment of age-related macular degeneration with photodynamic therapy (TAP) Study Group. Arch Ophthalmol. 1999;117(10):1329–45. 10532441

[3] Garg SJ, Hadziahmetovic M. Verteporfin Photodynamic Therapy for the Treatment of Chorioretinal Conditions: A Narrative Review. Clin Ophthalmol. 2024;18:1701–16. doi: 10.2147/OPTH.S464371 38881707 PMC11178081

[4] Sirks MJ, van Dijk EHC, Rosenberg N, Hollak CEM, Aslanis S, Cheung CMG, et al. Clinical impact of the worldwide shortage of verteporfin (Visudyne®) on ophthalmic care. Acta Ophthalmol. 2022;100(7):e1522–32. doi: 10.1111/aos.15148 35388619 PMC9790583

[5] Morishita T, Hayakawa F, Sugimoto K, Iwase M, Yamamoto H, Hirano D, et al. The photosensitizer verteporfin has light-independent anti-leukemic activity for Ph-positive acute lymphoblastic leukemia and synergistically works with dasatinib. Oncotarget. 2016;7(35):56241–52. doi: 10.18632/oncotarget.11025 27494842 PMC5302911

[6] Wang L-H, Baker NE. Salvador-Warts-Hippo pathway regulates sensory organ development via caspase-dependent nonapoptotic signaling. Cell Death Dis. 2019;10(9):669. doi: 10.1038/s41419-019-1924-3 31511495 PMC6739336

[7] Yu F-X, Zhao B, Guan K-L. Hippo Pathway in Organ Size Control, Tissue Homeostasis, and Cancer. Cell. 2015;163(4):811–28. doi: 10.1016/j.cell.2015.10.044 26544935 PMC4638384

[8] Liu-Chittenden Y, Huang B, Shim JS, Chen Q, Lee S-J, Anders RA, et al. Genetic and pharmacological disruption of the TEAD-YAP complex suppresses the oncogenic activity of YAP. Genes Dev. 2012;26(12):1300–5. doi: 10.1101/gad.192856.112 22677547 PMC3387657

[9] Wang C, Zhu X, Feng W, Yu Y, Jeong K, Guo W, et al. Verteporfin inhibits YAP function through up-regulating 14-3-3σ sequestering YAP in the cytoplasm. Am J Cancer Res. 2015;6(1):27–37. 27073720 PMC4759394

[10] Brodowska K, et al. The clinically used photosensitizer Verteporfin (VP) inhibits YAP-TEAD and human retinoblastoma cell growth in vitro without light activation. Experimental Eye Research 124, 67–73 (2014). 10.1016/j.exer.2014.04.011 PMC4135181 24837142

[11] Al-Moujahed A, Brodowska K, Stryjewski TP, Efstathiou NE, Vasilikos I, Cichy J, et al. Verteporfin inhibits growth of human glioma in vitro without light activation. Sci Rep. 2017;7(1):7602. doi: 10.1038/s41598-017-07632-8 28790340 PMC5548915

[12] Wei C, Li X. Verteporfin inhibits cell proliferation and induces apoptosis in different subtypes of breast cancer cell lines without light activation. BMC Cancer. 2020;20(1):1042. doi: 10.1186/s12885-020-07555-0 33121449 PMC7599100

[13] Dong L, Lin F, Wu W, Liu Y, Huang W. Verteporfin inhibits YAP-induced bladder cancer cell growth and invasion via Hippo signaling pathway. Int J Med Sci. 2018;15(6):645–52. doi: 10.7150/ijms.23460 29725256 PMC5930467

[14] Donohue E, Thomas A, Maurer N, Manisali I, Zeisser-Labouebe M, Zisman N, et al. The autophagy inhibitor verteporfin moderately enhances the antitumor activity of gemcitabine in a pancreatic ductal adenocarcinoma model. J Cancer. 2013;4(7):585–96. doi: 10.7150/jca.7030 24069069 PMC3781989

[15] Donohue E, Tovey A, Vogl AW, Arns S, Sternberg E, Young RN, et al. Inhibition of autophagosome formation by the benzoporphyrin derivative verteporfin. J Biol Chem. 2011;286(9):7290–300. doi: 10.1074/jbc.M110.139915 21193398 PMC3044985

[16] Donohue E, Balgi AD, Komatsu M, Roberge M. Induction of Covalently Crosslinked p62 Oligomers with Reduced Binding to Polyubiquitinated Proteins by the Autophagy Inhibitor Verteporfin. PLoS One. 2014;9(12):e114964. doi: 10.1371/journal.pone.0114964 25494214 PMC4262463

[17] Zhang H, Ramakrishnan SK, Triner D, Centofanti B, Maitra D, Győrffy B, et al. Tumor-selective proteotoxicity of verteporfin inhibits colon cancer progression independently of YAP1. Sci Signal. 2015;8(397):ra98. doi: 10.1126/scisignal.aac5418 26443705 PMC4818013

[18] Condurat A-L, Aminzadeh-Gohari S, Malnar M, Schider N, Opitz L, Thomas R, et al. Verteporfin-induced proteotoxicity impairs cell homeostasis and survival in neuroblastoma subtypes independent of YAP/TAZ expression. Sci Rep. 2023;13(1):3760. doi: 10.1038/s41598-023-29796-2 36882436 PMC9992669

[19] Hah Y-S, Han S-Y. Small-molecule-induced protein polymerization: mechanisms and therapeutic implications. Biomol Ther (Seoul). 2025;33:804–12. 10.4062/biomolther.2024.211 PMC12408205 40790935

[20] Lui JW, Xiao S, Ogomori K, Hammarstedt JE, Little EC, Lang D. The Efficiency of Verteporfin as a Therapeutic Option in Pre-Clinical Models of Melanoma. J Cancer. 2019;10(1):1–10. doi: 10.7150/jca.27472 30662519 PMC6329844

[21] Brouwer NJ, Konstantinou EK, Gragoudas ES, Marinkovic M, Luyten GPM, Kim IK, et al. Targeting the YAP/TAZ Pathway in Uveal and Conjunctival Melanoma With Verteporfin. Invest Ophthalmol Vis Sci. 2021;62(4):3. doi: 10.1167/iovs.62.4.3 33798262 PMC8024781

[22] Davies MJ. Singlet oxygen-mediated damage to proteins and its consequences. Biochem Biophys Res Commun. 2003;305(3):761–70. doi: 10.1016/s0006-291x(03)00817-9 12763058

[23] Krieg M, Whitten DG. Self-sensitized photooxidation of protoporphyrin IX and related free-base porphyrins in natural and model membrane systems. Evidence for novel photooxidation pathways involving amino acids. J Am Chem Soc. 1984;106:2477–9.

[24] Fancy DA, Kodadek T. Chemistry for the analysis of protein-protein interactions: rapid and efficient cross-linking triggered by long wavelength light. Proc Natl Acad Sci U S A. 1999;96(11):6020–4. doi: 10.1073/pnas.96.11.6020 10339534 PMC26828

[25] Fancy DA, Denison C, Kim K, Xie Y, Holdeman T, Amini F, et al. Scope, limitations and mechanistic aspects of the photo-induced cross-linking of proteins by water-soluble metal complexes. Chem Biol. 2000;7(9):697–708. doi: 10.1016/s1074-5521(00)00020-x 10980450

[26] Jiang S, Carroll L, Mariotti M, Hägglund P, Davies MJ. Formation of protein cross-links by singlet oxygen-mediated disulfide oxidation. Redox Biol. 2021;41:101874. doi: 10.1016/j.redox.2021.101874 33601275 PMC7900768

[27] Konstantinou EK, Notomi S, Kosmidou C, Brodowska K, Al-Moujahed A, Nicolaou F, et al. Retraction Note: Verteporfin-induced formation of protein cross-linked oligomers and high molecular weight complexes is mediated by light and leads to cell toxicity. Sci Rep. 2024;14(1):11040. doi: 10.1038/s41598-024-61893-8 38744880 PMC11093967

[28] Read RD. Repurposing the drug verteporfin as anti-neoplastic therapy for glioblastoma. Neuro Oncol. 2022;24(5):708–10. doi: 10.1093/neuonc/noac019 35100422 PMC9071275

[29] Lao Z, Chen X, Pan B, Fang B, Yang W, Qian Y. Pharmacological regulators of Hippo pathway: Advances and challenges of drug development. FASEB J. 2025;39(6):e70438. doi: 10.1096/fj.202401895RR 40100056

[30] Ochsner M. Photophysical and photobiological processes in the photodynamic therapy of tumours. J Photochem Photobiol B. 1997;39(1):1–18. doi: 10.1016/s1011-1344(96)07428-3 9210318

[31] Dědic R, Kořínek M, Molnár A, Svoboda A, Hála J. Singlet oxygen quenching by oxygen in tetraphenyl-porphyrin solutions. Journal of Luminescence. 2006;119–120:209–13. doi: 10.1016/j.jlumin.2005.12.032

[32] Mesrouze Y, Bokhovchuk F, Meyerhofer M, Fontana P, Zimmermann C, Martin T, et al. Dissection of the interaction between the intrinsically disordered YAP protein and the transcription factor TEAD. Elife. 2017;6:e25068. doi: 10.7554/eLife.25068 28430104 PMC5400505

[33] Anbanandam A, Albarado DC, Nguyen CT, Halder G, Gao X, Veeraraghavan S. Insights into transcription enhancer factor 1 (TEF-1) activity from the solution structure of the TEA domain. Proc Natl Acad Sci U S A. 2006;103(46):17225–30. doi: 10.1073/pnas.0607171103 17085591 PMC1859914

[34] Gibault F, et al. Non-Photoinduced Biological Properties of Verteporfin. CMC 2016;23:1171–84 10.2174/0929867323666160316125048 26980565

[35] Baroja I, Kyriakidis NC, Halder G, Moya IM. Expected and unexpected effects after systemic inhibition of Hippo transcriptional output in cancer. Nat Commun. 2024;15(1):2700. doi: 10.1038/s41467-024-46531-1 38538573 PMC10973481

[36] Read W. A phase 1 / 2 study of Visudyne (liposomal verteporfin) in persons with recurrent high grade EGFR-mutated glioblastoma. 2025. https://clinicaltrials.gov/study/NCT04590664

[37] Yap TA, Kwiatkowski DJ, Dagogo-Jack I, Offin M, Zauderer MG, Kratzke R, et al. YAP/TEAD inhibitor VT3989 in solid tumors: a phase 1/2 trial. Nat Med. 2025;31(12):4281–90. doi: 10.1038/s41591-025-04029-3 41111090

