# Supplementary material for: Ambient light drives verteporfin-induced protein cross-linking during standard laboratory sample processing
Source: PLoS One. 2026 Sep 21;21(9):e0358654. doi: 10.1371/journal.pone.0358654 (PMC13592631; doi:10.1371/journal.pone.0358654)
Supplement: Supplementary file 1 [file pone.0358654.s001.pdf]

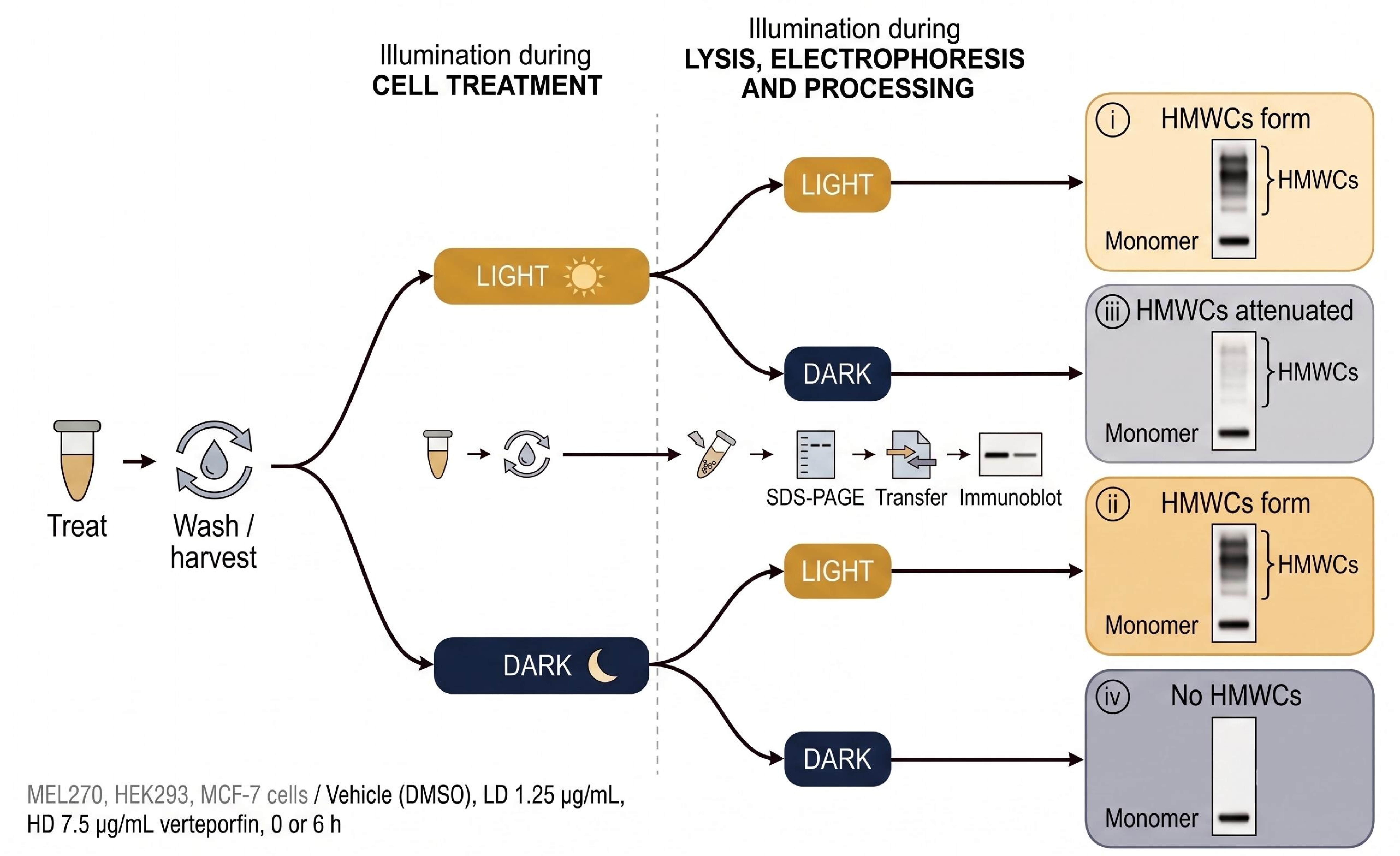
Illumination during
CELL TREATMENT
Illumination during
LYSIS, ELECTROPHORESIS
AND PROCESSING
Treat
Wash /
harvest
LIGHT
DARK
LIGHT
DARK
LIGHT
DARK
SDS-PAGE
Transfer
Immunoblot
i HMWCs form
HMWCs
Monomer
iii HMWCs attenuated
HMWCs
Monomer
ii HMWCs form
HMWCs
Monomer
iv No HMWCs
Monomer
MEL270, HEK293, MCF-7 cells / Vehicle (DMSO), LD 1.25 µg/mL,
HD 7.5 µg/mL verteporfin, 0 or 6 h
