# Supplementary material for: Ambient light drives verteporfin-induced protein cross-linking during standard laboratory sample processing
Source: PLoS One. 2026 Sep 21;21(9):e0358654. doi: 10.1371/journal.pone.0358654 (PMC13592631; doi:10.1371/journal.pone.0358654)
Supplement: Supplementary file 2 [file pone.0358654.s002.docx]

**Supplementary Table 1. Antibody Details**

Antibodies used for Western blot analysis in this study.

| **Antibody** | **Host** | **Dilution** | **Catalog #** | **RRID** | **Company** |
| --- | --- | --- | --- | --- | --- |
| Anti-p62/SQSTM1 | Rabbit | 1:1000 | #5114 | AB_10624872 | Cell Signaling Technology |
| Anti-DIAP1 | Rabbit | 1:1000 | #5486 | AB_10828440 | Cell Signaling Technology |
| Anti-ROCK1 (C8F7) | Rabbit | 1:1000 | #4035 | AB_1549585 | Cell Signaling Technology |
| Anti-YAP | Rabbit | 1:1000 | #4912 | AB_2218911 | Cell Signaling Technology |
| Anti-phospho-YAP (Ser127) | Rabbit | 1:1000 | #4911 | AB_2218913 | Cell Signaling Technology |
| Anti-TEF1/TEAD1 [EPR3967(2)] | Rabbit | 1:1000 | #ab133533 | AB_2737294 | Abcam |
| Anti-methionine sulfoxide | Rabbit | 1:200 | #600166 | AB_3740087 | Cayman Chemical |
| Anti-β-actin (13E5) | Rabbit | 1:2000 | #4970 | AB_2223172 | Cell Signaling Technology |
| HRP-linked anti-rabbit IgG | Goat | 1:2000 | #7074 | AB_2099233 | Cell Signaling Technology |
